# Short-Term Effect of Spiritual Music on Heart Rate Variability in Medical Students: A Single-Group Experimental Study

**DOI:** 10.7759/cureus.34833

**Published:** 2023-02-10

**Authors:** Kavin Adlakha, Mohit K Mathur, Anjum Datta, Rupali Kalsi, Bharti Bhandari

**Affiliations:** 1 Medical School, Government Institute of Medical Sciences (GIMS), Greater Noida, IND; 2 Department of General Surgery, Government Institute Of Medical Sciences (GIMS), Greater Noida, IND; 3 Department of Physiology, Army College of Medical Sciences, Delhi, IND; 4 Department of Dentistry, Government Institute of Medical Sciences (GIMS), Greater Noida, IND; 5 Department of Physiology, Government Institute of Medical Sciences (GIMS), Greater Noida, IND

**Keywords:** medical students, blood pressure, stress, heart rate variability, om chanting, spiritual music

## Abstract

Background: There are ancient texts and modern studies alluding to the therapeutic benefits obtained from listening to music. Studies have shown that chanting "OM" has a relaxing effect by causing parasympathetic dominance, limbic deactivation, and decreasing the brain's dopamine levels. This research aims to study the effect of listening to OM chanting on the cardiovascular system and heart rate variability and its possible use as a stress buster among medical students.

Materials and Methods: Fifty medical undergraduates were selected for the study. After a 20-minute relaxation, a lead 2 electrocardiogram (EKG) was recorded for 10 minutes. Their blood pressure (BP) and heart rate were measured. The subjects were then made to listen to OM chanting for 20 minutes, immediately after which their BP and heart rate were measured. This was followed by another 10-minute lead 2 EKG. The EKGs recorded were then used to calculate the standard deviation in N-N interval (SDNN), total power, high-frequency power, and low-frequency power.

Results: The study reported a significant decrease in blood pressure and heart rate and a significant increase in SDNN and total power. There was also an insignificant increase in low frequency and an insignificant decrease in high frequency.

Conclusion: This study provides insight into the importance of spiritual music therapy in the maintenance of mental as well as cardiovascular health among medical students.

## Introduction

Evidence pertaining to the therapeutic benefits obtained from listening to music can be found in ancient Greek literature. Noteworthy scientists included some great names like Pythagoras and Aristotle [1]. The middle ages were associated with the emergence of detailed studies exploring the beneficial effects of listening to music. Multiple interventions performed on athletes have shown that both slow and fast tempos of music have led to better performance results when compared to those in the control group. Listening to music has a slow tempo and minimal dynamic contrasts have been found to be effective in decreasing the heart rate, respiratory rate, and blood pressure [1].

Thus, following the same lines, it can be hypothesized that listening to "OM" chanting, either at a frequency of 432hz or 528hz, should have a relaxing effect. In fact, studies done with various neuroimaging techniques like functional magnetic resonance imaging (fMRI), functional near-infrared spectroscopy (fNIRS), electroencephalogram (EEG), middle latency potential response, and auditory evoked potential response have suggested a shift to parasympathetic system dominance, enhanced cardiopulmonary efficiency, increased attention, and reduced complexity of EEG signals [2]. OM meditation is also expected to lower the blood pressure and the heart rate of the subject [3], regulating the rhythm and thus providing cardiovascular benefits.

Heart rate variability (HRV), as the name suggests is the variation in the heart rate of an individual. More precisely, HRV is the minute fluctuations in the time interval (typically tens of milliseconds long) between successive heartbeats or in an electrocardiogram (EKG), two adjacent Q-Q (or N-N) intervals. Generally speaking, low HRV is a bad omen, either an indicator of a currently underlying disorder or a predictor of a future health problem [4]. Low HRV is usually seen in people having higher resting heart rates or those suffering from pathological conditions such as diabetes, inflammation, insomnia, asthma, anxiety, and depression [5].

However, higher HRV may be a cause of concern when the cause is pathological and not physiological in nature, e.g., elevated HRV due to cardiac abnormality, which causes increased mortality in elders except for increased survival. Thus, an optimal level of HRV, generated physiologically, such as a higher level of vagally-generated HRV, is a measure of the resilience of the heart and is found to have a positive correlation with executive functions like attention and emotional processing [5].

Studies have been done focusing on the health benefits and improving the performance of athletes with music therapy [1]. Furthermore, there is available literature stating the beneficial effects of spiritual music on reducing stress scores by upregulation of parasympathetic activity [6]. Evidence exists that medical students experience high levels of stress owing to strenuous medical programs and long working hours. The estimated prevalence of emotional stress and psychological morbidity is higher in medical students than in the general population [7]. It can be hypothesized that spiritual therapy may have beneficial effects on the mental and emotional well-being of medical undergraduates. Thus, this study was undertaken with the aim to evaluate the short-term effect of listening to OM chants on cardiovascular and neurocardiac functioning and its possible use as a stress-buster among medical students.

## Materials and methods

This was a single-group experimental study conducted in Government Institute of Medical Sciences, Greater Noida, Uttar Pradesh, India. Fifty medical students between the age of 18 and 25 years were included in the study. Students with smoking and/or drinking habits, physical disabilities, congenital anomalies, psychiatric disorders, history of chronic illness and/or medication for the same, and self-reported disturbed circadian cycle were excluded from the study. Signed informed consent was taken from the participants after they had been through the information sheet that explained to them the aim, procedure, and potential risks that were involved with this study.

The study was given ethical clearance from the Institutional Ethics Committee, Government Institute of Medical Sciences, Greater Noida, India (Approval number: GIMS IEC-ECR/1224/Inst./UP/2019). The study was conducted between 9 AM and 1 PM. The participants were tested in a well lit-room, at a temperature that the subject was comfortable with (20-25ºC). The subjects were advised to keep their eyes closed. The participants were advised not to have meals or consume caffeinated drinks two hours prior to reporting for the study. The participants were made to rest for 20 minutes, after which their lead 2 EKG was recorded using PowerLab (ADInstruments, Sydney, Australia). After the EKG recording, participants were made to listen to OM chanting for 20 minutes. The music was delivered via headphones at 60-70 dB. The frequency of OM chanting selected for this study was 528 Hz. This was followed by another 10-minute EKG recording.

Standard deviation in Q-Q wave (SDNN1), total power (TP), contribution of high frequency to total power (n-HF), contribution of low frequency to total power (n-LF), and the ratio of n-LF to n-HF (LF:HF) were calculated using LabChart v 8.0 (ADInstruments, Sydney, Australia). Heart rate was manually measured and blood pressure was measured using a sphygmomanometer immediately before and immediately after music therapy. 

Recorded parameters

Sociodemographic Variables

Age, weight, height, and gender of the subjects were the sociodemographic variables collected. The body mass index (BMI) of the subjects was calculated by Quetelet’s index (ratio of weight in kilograms and square of height in meters squared).

HRV

HRV was calculated using LabChart v 8.0 by following the procedure recommended by the European Task Force study [5]. The HRV was calculated for 10 minutes before and after the music therapy. HRV was assessed in two ways: (i) Time domain (SDNN1) and (ii) Frequency domain (n-HF, n-LF, LF:HF, TP).

Physiological Parameters

Systolic blood pressure and diastolic blood pressure were measured by a sphygmomanometer and heart rate was measured manually immediately before and after the music therapy.

Data management and statistical analysis

Data entry was done in Microsoft Excel (Microsoft Corporation, Redmond, Washington, United States) and statistical analysis was done using IBM SPSS Statistics for Windows, Version 28.0 (Released 2021; IBM Corp., Armonk, New York, United States). Paired student t-test was applied to test the statistical significance of the obtained result.

## Results

Demographic data

A total of 50 participants comprising 30 males and 20 females with a mean age of 20.98 years (SD= 1.27) were recruited for the study. The mean BMI for males was 22.36 (SD=3.20) and for females, it was 21.87 (SD=2.99) (Table 1).

**Table 1:** Demographic profile of subjects

Information	Male (Mean ± SD)	Female (Mean ± SD)	Overall (Mean ± SD)
Sample Size (n)	30	20	50
Age (years)	21.17±1.37	20.7±1.08	20.98±1.27
Weight (kg)	68.37±10.73	56.55±6.38	63.64±11.25
Height (cm)	174.79±6.62	160.93±6.38	169.24±9.42
BMI (kg/m^2^)	22.36±3.20	21.87±2.99	22.17±3.10

Pre-music therapy and post-music therapy comparison

Time Domain Comparison

The time domain analysis of the ECG for SDNN showed a statistically significant increase in pre- and post-music therapy. Before music therapy, the mean SDNN found was 64.34 msec (SD=23.26) and after music therapy, the mean increased to 73.54 msec (SD=26.62). The difference was statistically significant with a p-value less than 0.001 (Table 2).

**Table 2:** Pre-music therapy and post-music therapy comparison SDNN: standard deviation in N-N interval; n-LF: power contributed by low frequency; n-HF: power contributed by high frequency p <0.05 is considered significant

Parameters	Pre-Music (Mean ± SD)	Post-Music (Mean ± SD)	Difference	t-score	p-value
Systolic Blood Pressure (mmHg)	112.76±9.29	107.68±8.33	-5.08	-8.971	<0.001
Diastolic Blood Pressure (mmHg)	70.56±9.04	65.04±7.17	-5.52	-7.440	<0.001
Heart Rate (BPM)	70.66±9.86	68.26±8.74	-2.4	-4.733	<0.001
SDNN (msec)	64.35±23.26	73.54±26.62	9.19	4.827	<0.001
n-LF	43.57±18.70	45.13±18.55	1.56	1.037	0.305
n-HF	54.69±17.30	53.32±17.41	-1.36	-0.920	0.362
Total Power (msec^2^)	4900.39±4520.76	6252.64±5063.15	1352.25	3.178	0.003
LF:HF	1.039±0.830	1.112±0.878	0.073	0.916	0.364

Frequency Domain Comparison

The frequency domain analysis for n-HF, n-LF, LF:HF, and TP showed insignificant changes for HF, LF, and LF:HF but significant changes in TP. The pre-music therapy mean of n-HF was 54.69 (SD=17.30) and the post-music therapy mean of n-HF was lower at 53.32 (SD=17.41). This difference, however, was statistically insignificant (p-value=0.362) (Table 2). Pre-music therapy mean of n-LF was 43.57 (SD=18.70). There was an increase in n-LF values to 45.13 (SD=18.55). This difference again, however, was statistically insignificant (p-value=0.305). The pre-music therapy mean for TP was 4900.394 msec^2^ (SD=4520.76). There was a significant increase in mean of TP after music therapy. The post-music therapy mean of TP was 6252.64 msec^2^ (SD=5063.15) and the p-value was 0.003. The LF: HF ratio was 1.039 (SD=0.830) pre-music therapy. This ratio was increased to 1.112 (SD=0.878) post-music therapy. The difference was insignificant (p-value=0.364) (Table 2)

Cardiovascular Parameter Comparison

BP: The mean systolic BP before music therapy was 112.76 mmHg (SD=9.29) and it reduced to 107.68 (SD=8.33). This difference was statistically significant (p-value<0.001) (Table 2). The mean diastolic BP also showed the same trend. The pre-music therapy mean of diastolic BP was 70.56 mmHg (SD=9.04) and post-music diastolic BP reduced to 65.04 mmHg (SD=7.17). This difference was also statistically significant (p-value<0.001) (Table 2)

Heart Rate: Similarly for heart rate, the pre-music therapy mean of heart rate was 70.66 bpm (SD=9.86). Post music therapy, the mean decreased to 68.26 bpm (SD=8.74). These results were statistically significant (p-value<0.001) (Table 2).

## Discussion

In the present study, the effect of spiritual music and its possible association with cardiovascular parameters and HRV was assessed in 50 undergraduate medical students, comprising 60% males and 40% females. The mean age of participants was 20.98±1.27 years.

Spiritual music has a multitude of health benefits. Evidence suggests that listening to soothing spiritual music calms the mind, reduces anxiety, and brings positive changes in HRV and heart rate [8]. Cardiovascular health can be assessed with numerous parameters, viz. EKG, emission tomography (ET-Scan), MRI, etc. EKG is a non-invasive, easy-to-perform test to check for electrical activity of the heart. The parameters recorded were BP, heart rate, SDNN, n-HF, n-LF, LF:HF, and TP.

Monitoring of systolic and diastolic BP gives a near-to-clear picture of heart health. In the present study, post music therapy, 90% of subjects showed a decrease in systolic BP, 84% showed a decrease in diastolic BP, 78% showed a reduction in heart rate and 54% showed a reduction in all three parameters. This drop in BP and heart rate post music therapy is suggestive of psychophysiological relaxation representing a reduction in stress and anxiety [3,9]. This can be attributed to the fact that the sound of OM chants induces deep relaxation in the body and mind. The decrease in sympathetic activity and upregulation of parasympathetic activity due to an increase in the brain's dopamine levels after listening to spiritual music brings about a decrease in the heart rate and thus systolic BP [10].

A similar study done by Loomba et al. showed that music therapy decreases systolic BP and diastolic BP [9]. Conflicting evidence was reported by Mir et al., who reported that music therapy had an insignificant effect on diastolic BP [10]. However, an important point to note is that in these studies the music therapy was given regularly for many weeks (25 minutes daily for four weeks [9] and 30 minutes for five days a week for four weeks [10]) while this study dealt with the immediate effect of listening to spiritual music.

Time domain indices of HRV like SDNN, root mean square of successive differences (RMSSD), posterior nasal nerve (pNN50%), etc. are used to quantify the variabilities seen in inter-beat intervals. The frequency domain is used to estimate the distribution of variance as a function of frequency [11]. These frequencies refer to the frequencies of heart rate oscillations, which are classified as ultra-low frequencies, very low frequencies, low frequencies, and high frequencies. The sum of all these frequencies is equal to TP. In the case of short-term recordings, only TP, n-LF, and n-HF assessments can be made [11].

TP is the variance while SDNN refers to the SD (i.e., square root of variance) in the duration of successive heartbeats over a specified period of time. Thus, the factors that cause a change in SDNN value will also cause a change in TP in the same direction. Thus, conditions such as sympathetic activation are usually noted with a decrease in SDNN and TP, whereas TP is seen to increase in cases with vagal nerve stimulation.

In a resting healthy individual in the supine position, the majority of SDNN is associated with the activity of the parasympathetic system [12]. Thus, the increase in SDNN and TP provides evidence of vagal nerve stimulation. This agrees with the findings of Kalyani et al., who in 2011 found that OM chanting resulted in the deactivation of the limbic region [13], which is seen during transcutaneous vagal nerve stimulation therapy given to patients suffering from depression, anxiety, and epilepsy [14]. Thus, it can be hypothesized that the effectiveness of music therapy is related to its ability to modulate autonomic nervous system towards parasympathetic dominance by vagal nerve stimulation via its auricular branches [13]. However, a study done by Inbaraj et al. found insignificant changes in SDNN values in yoga practitioners after they underwent a five-minutes OM chanting session [15]. However, the discrepancy in the findings might be because the subjects were exposed to OM chanting for a longer period in this study.

n-LF is the power in the frequency range of 0.04-0.15 Hz [11]. It is used as a marker of baroreflex and sympathetic nervous system activity [1]. However, under resting conditions, the LF band can be used as a measure of baroreflex activity [4]. In our study, a trend of increase in n-LF was observed after music therapy, though the difference was statistically non-significant. Increased baroreflex activity can explain the significant decrease in systolic BP and heart rate found in this study. The statistical insignificance of this parameter may be due to low sample size or more likely due to the short duration of exposure to music therapy as another study done by Kachanathu et al. in athletes found a significant decrease in n-LF after 29 days of music therapy (five minutes each day) [1]. Another study that had a shorter duration of exposure to OM chanting (five-minute exposure) done by Inbaraj et al. reported insignificant changes in low frequency [15].

n-HF or the respiratory band, named so because it reflects the HRVs caused by the respiratory cycle, refers to the power in the frequency range of 0.15-0.4 Hz [11]. The main modulator of n-HF is respiratory sinus arrhythmia. Slow, deep breathing can increase heart rate fluctuations [5] and cause a significant increase in n-HF, as noted in studies done to assess the effect of OM chanting in yoga practitioners [15].

In the current study, the subjects were made to listen to OM chanting rather than do OM chanting. This might be the reason that changes in the n-HF band are found to be insignificant. A significant decrease in cardiovascular parameters was observed while a significant increase in SDNN and TP was seen. The following might be the possible explanation/explanations for the observed results: (i) Increase in brain dopamine levels leading to a decrease in sympathetic activity and an increase in parasympathetic activity; (ii) vagal nerve stimulation via auricular branches causing limbic deactivation and modulation of autonomic activity towards parasympathetic dominance; (iii) increased activity of baroreflex causing a significant reduction in systolic BP and heart rate.

Lesser sample size and lack of estimation of the stress hormone cortisol are some of the study's limitations.

## Conclusions

Medicine is among the highest competitive professions. The stress and anxiety associated with this field can even be felt by those who have not yet entered the profession. The study thus provides insight into the potential use of spiritual music therapy in the maintenance of cardiovascular health and mental health among medical students. The results of this study show that music therapy is possibly an effective way of reducing stress and its calming effect can help to lower blood pressure. Future studies with larger sample sizes, stringent criteria for patient selection, quantification of stress scores, and longer follow-up should be carried out to substantiate the results of our study.
